# Variability of residents’ ratings of faculty’s teaching performance measured by five- and seven-point response scales

**DOI:** 10.1186/s12909-020-02244-9

**Published:** 2020-09-22

**Authors:** Maarten P. M. Debets, Renée A. Scheepers, Benjamin C. M. Boerebach, Onyebuchi A. Arah, Kiki M. J. M. H. Lombarts

**Affiliations:** 1 Amsterdam Center for Professional Performance and Compassionate Care, Department of Medical Psychology, Amsterdam UMC, University of Amsterdam, Meibergdreef 9, PO Box 22700, 1100 DE Amsterdam, The Netherlands; 2 Research group Socio-Medical Sciences, Erasmus School of Health Policy and Management, Erasmus University of Rotterdam, Rotterdam, The Netherlands; 3 Department of Epidemiology, Fielding School of Public Health, University of California, Los Angeles (UCLA), Los Angeles, California USA; 4 UCLA Center for Health Policy Research, Los Angeles, California USA; 5 Center for Social Statistics, UCLA, Los Angeles, California USA; 6 Department of Statistics, UCLA, Los Angeles, California USA

**Keywords:** Teaching performance, Performance ratings, Response categories, Residents, Faculty, Five-point scale, Seven-point scale, Variability

## Abstract

**Background:**

Medical faculty’s teaching performance is often measured using residents’ feedback, collected by questionnaires. Researchers extensively studied the psychometric qualities of resulting ratings. However, these studies rarely consider the number of response categories and its consequences for residents’ ratings of faculty’s teaching performance. We compared the variability of residents’ ratings measured by five- and seven-point response scales.

**Methods:**

This retrospective study used teaching performance data from Dutch anaesthesiology residency training programs. Questionnaires with five- and seven-point response scales from the extensively studied System for Evaluation of Teaching Qualities (SETQ) collected the ratings. We inspected ratings’ variability by comparing standard deviations, interquartile ranges, and frequency (percentage) distributions. Relevant statistical tests were used to test differences in frequency distributions and teaching performance scores.

**Results:**

We examined 3379 residents’ ratings and 480 aggregated faculty scores. Residents used the additional response categories provided by the seven-point scale – especially those differentiating between positive performances. Residents’ ratings and aggregated faculty scores showed a more even distribution on the seven-point scale compared to the five-point scale. Also, the seven-point scale showed a smaller ceiling effect. After rescaling, the mean scores and (most) standard deviations of ratings from both scales were comparable.

**Conclusions:**

Ratings from the seven-point scale were more evenly distributed and could potentially yield more nuanced, specific and user-friendly feedback. Still, both scales measured (almost) similar teaching performance outcomes. In teaching performance practice, residents and faculty members should discuss whether response scales fit their preferences and goals.

## Background

In many residency training programs, faculty’s teaching performance evaluation is part of continuous efforts to maintain or enhance teaching quality [1–3]. Often, to gain insight into the strengths and weaknesses of faculty’s teaching performance, feedback from residents is collected using questionnaires [1, 2, 4, 5]. Residents’ feedback informs summative and formative purposes such as faculty development, promotion, appointment and remuneration [2, 3, 6]. Therefore, it is crucial that questionnaires measuring faculty’s teaching performance are valid, reliable, and fit its practical use.

Researchers well-investigated classic measures of reliability and validity of questionnaires, for example, by calculating Cronbach’s alphas and correlations between other measures that should be associated theoretically [4, 7–10]. However, the number of response categories is an important validity aspect of questionnaires that these studies rarely consider [4, 7–9, 11]. The number of response categories might affect residents’ ratings of faculty’s performance (e.g. means, frequency distributions) [12–17].

In the setting of teaching performance evaluation, the purpose of response scales is to measure residents’ perceptions about faculty’s functioning [4, 18, 19]. Residents usually rate multiple faculty members with whom they sometimes have long-term and vulnerable relationships [1, 18–20]. Moreover, response scales must be able to reflect residents’ perceptions about the performances of different faculty members. Residents’ that feel too restricted by the response categories may find the questionnaire less user-friendly [16, 21] and could be less willing to rate faculty’s performance. Also, this might lead to discriminative performance information to be lost [16, 22]. Faculty members might value the additional performance information as it is more specific [3, 20, 23] and can more precisely inform improvement directions.

When determining the optimal number of response categories, a trade-off arises between maximizing the information transmission and limiting respondent demands [15, 24, 25]. A scale that presents too few response categories fails to discriminate between respondents with different perceptions and yields a reduced amount of information [24, 25]. Too many response categories can opaque the meaning of response options, making it hard for respondents to differentiate between options [24, 25]. Psychometric qualities usually seem to increase up to seven response categories [15, 24, 26–28], which is also how many options most respondents can differentiate [29].

Various response scale formats have different properties (e.g. ease of use, preference for expanded interval) being more or less suitable for a certain measurement context [15, 25, 28]. Therefore, questionnaire developers should appraise the appropriateness of the number of response categories within its specific measurement context, including the population and object under investigation [24, 25, 28].

Most questionnaires evaluating faculty’s teaching performance use five-point Likert scales [4, 9]. Various studies indicate that five-point response scales do not fit respondents’ discriminative capacity for subjective measures like teaching performance [16, 21, 22]. More specifically, residents are cognitively and verbally skilled and often experienced with conducting questionnaires – factors contributing to their ability to differentiate between more than five gradations of teaching performance [24]. Furthermore, faculty’s teaching performance evaluations usually show skewness towards the positive scale spectrum [14]. On a five-point Likert scale, this means that residents only have two response options to differentiate between good performances.

Literature suggests that seven-point scales reflect residents’ perceptions of faculty’s teaching performance more adequately compared to five-point scales, without harming or even improving psychometric qualities [15, 16, 22, 30]. Switching from five to seven response categories might benefit residents and teaching faculty. However, given the summative and formative purposes of teaching performance feedback, decisions to adjust response scales require justification. Inspecting the variability of performance ratings can provide insight in how residents use different response categories.

Therefore, the purpose of this study was to determine whether the findings on the statistical properties (utility and validity) of five- versus seven-point response scales are replicable in questionnaires used for evaluating teaching performance in graduate medical education. To this end, we compared the variability of residents’ ratings of faculty’s teaching performance using five- and seven-point response scales. We additionally examined whether both scales resulted in similar teaching performance outcomes. Based on prior research, we expected that residents would use the additional — especially the positive — response categories of the seven-point scale [14, 16]. Also, we expected no substantial differences in ratings’ mean scores and standard deviations (after rescaling) [12, 13]. This study should be seen as part of the foundation for continuing research in this domain, with particular focus on continuously understanding and improving the validity, reliability and utility of teaching performance evaluation questionnaires.

## Methods

### Study design, setting and population

This retrospective study is part of the ongoing large-scale evaluation and improvement of teaching performance conducted among anaesthesiology training programs in the Netherlands [18, 19]. The residency training takes place in both university medical centres and affiliated general teaching hospitals. Faculty members train anaesthesiology residents for up to 6 years. The Dutch accrediting body for residency training programs prescribes that supervisors ask for feedback from their residents. In the Netherlands, the most widely used system to organise and collect this feedback is the System for Evaluation of Teaching Qualities (SETQ), which includes both resident- and faculty-completed questionnaires to evaluate faculty’s teaching performance. The SETQ data, which include both scored evaluations and narrative feedback, are routinely used for formative purposes to improve faculty teaching performance [1, 5, 14, 18, 19]. Ethical approval was waived by the institutional ethical review board of the Academic Medical Center of the University of Amsterdam.

### Instruments

The SETQ was first developed in 2008 to evaluate the teaching performance of anaesthesiology faculty members, followed by speciality-specific SETQ questionnaires for medical specialties [1], surgical specialities [5], and obstetrics and gynaecology [31]. SETQ data used in this study consist of anaesthesiology residents’ ratings of their faculty.

The original SETQ questionnaire contains 22 core items capturing five domains of teaching quality, namely: ‘learning climate’, ‘professional attitude towards residents’, ‘communication of goals’, ‘evaluation of residents’ and ‘feedback’ [18]. Residents answer all items on a five-point Likert scale (‘totally disagree’, ‘disagree’, ‘neutral’, ‘agree’, and ‘totally agree’). A modernised version of the SETQ was validated for anaesthesiology training programs in 2013, resulting in the SETQ *smart*. The SETQ *smart* contains twelve identical core items from the original SETQ (Table 1). Other items were (slightly) adjusted or new. Based on input from residents, the SETQ *smart* uses a seven-point response scale (‘totally disagree’, ‘somewhat disagree’, ‘disagree’, ‘neutral’, ‘somewhat agree’, ‘agree’, and ‘totally agree’) [19]. In the rest of this study, we refer to the SETQ and SETQ *smart* as the five- and seven-point questionnaire, respectively. The Additional file 1 presents the templates of both questionnaires.

**Table 1:** Identical five- and seven-point questionnaire items compared in this study

1. *Encourages residents to participate actively in discussions*	
2. *Stimulates residents to bring up problems*	
3. *Motivates residents to study further*	
4. *Stimulates residents to keep up with the literature*	
5. *Prepares well for teaching presentations and talks*	
6. *Listens attentively to residents*	
7. *Is respectful towards residents*	
8. *Is easily approachable during on-calls*	
9. *Evaluates residents’ specialty knowledge regularly*	
10. *Evaluates residents’ analytical abilities regularly*	
11. *Gives corrective feedback to residents*	
12. *Offers suggestions for enhancement*	
Total score (TS)^a^	

^a^Mean average of the 12 identical items

### Data collection

This study used data from Dutch anaesthesiology training programs collected between January 2013 and January 2017. Representatives of the training programs could choose to use the seven-point questionnaire when available. Data were collected using a password protected online platform, which was developed specifically for facilitating physicians’ performance evaluations. Invitations were emailed to residents through the platform on the first day of the data collection period, stressing confidential and anonymous participation. The emails contained personal passwords enabling protected and safe personal login. For each training program, data collection usually lasted four to 6 weeks, i.e. one measurement period. Residents could participate in multiple measurement periods. During measurement periods residents evaluated one to multiple faculty members. Up to three reminders were sent to non-responders. Immediately after closure of the data collection period, all teaching faculty members could download their feedback reports.

### Analysis

Residents’ ratings containing more than 50% missing values were excluded from our dataset, remaining missing values were imputed using expectation maximization (EM). Descriptive statistics were used to summarise the characteristics of residents and their ratings in the five- and seven-point questionnaire samples. All analyses were performed using the identical items of the five- and seven-point questionnaire (Table 1).

We compared various indicators of variability – standard deviations, interquartile ranges (IQRs), and frequency (percentage) distributions – of residents’ ratings and aggregated faculty scores. Aggregated faculty scores were calculated by aggregating residents’ ratings of a particular faculty member with three or more ratings – for reliable domain and overall scores – from one measurement period to the mean [32]. Frequency percentages were calculated for the response categories presented in both scales: ‘totally disagree’, ‘neutral’ and ‘totally agree’. Also, we counted how often residents used the other response categories in each questionnaire. Furthermore, we calculated the percentage of ratings above and below the ‘neutral’ response category for each questionnaire. Next, to assess whether frequencies (or response category percentages) of similar categories and percentages below and above the ‘neutral’ category were dependent on the number of response categories, i.e. five and seven, we performed chi-square tests. For each item, chi-square tests compared frequencies of the four categories (‘totally disagree’, ‘neutral’, ‘totally agree’, ‘other categories’) for the five- and seven-point questionnaire. Chi-square tests were also used to compare the proportions for scoring on the ‘neutral’ category and below and above this category (i.e. 2 × 3 contingency table). To assess differences in response category percentages and percentages of ratings below and above the ‘neutral’ category, we conducted post hoc testing using adjusted standardised residuals [33, 34]. To control for multiple comparisons, we used the Bonferroni procedure.

To check whether both scales measured the same teaching performance outcomes, we compared rescaled means and standard deviations of residents’ ratings and aggregated faculty scores of the five- and seven-point questionnaire. For rescaling five-point scale ratings to match those from the seven-point scale, we used the formula: y = 1.5x – 0.5. In the formula, ‘x’ represents the original rating of the five-point scale and ‘y’ the transformed score. After rescaling, we performed 13 independent samples t-tests on residents’ ratings and aggregated faculty scores to test differences in the means of identical items of both questionnaires. We used Levene’s test to assess whether variances could be assumed equal. We corrected for multiple comparisons using the Bonferroni procedure.

To adjust for non-random assignment of residents to the five- and seven-point questionnaires, we repeated our independent samples t-tests using a selection weight. We calculated inverse probability of selection (response) weights for residents’ ratings and aggregated faculty scores [35]. This procedure matches the sample characteristics of both questionnaires by multiplying scores by a calculated weight. Weights for residents’ ratings were based on the type of hospital (academic vs non-academic), sex, year of residency training, and scores on all identical items of the five- and seven-point questionnaires. For aggregated faculty scores, weights were based on the type of hospital, the number of residents’ ratings per measurement period, aggregated scores of all indentical items and the questions measuring faculty’s overall teaching performance (Additional file 1). Cuttoff weight values were > 5 and < .2, meaning all residents’ ratings and aggregated faculty scores weighing more than 5 and less than .2 were assigned a weight of .2 and 5 respectively.

SPSS was used for all analyses (IBM Corp. Released 2016. IBM SPSS Statistics for Windows, Version 24.0. Armonk, NY: IBM Corp.).

## Results

### Study participants and description of the samples

In total, data from 44 ratings (1.3% of total) were excluded from our dataset due to more than 50% missing values: 36 from the five-point questionnaire and 8 from the seven-point questionnaire. Table 2 presents a description of the study participants and ratings of both questionnaire samples.

**Table 2:** Characteristics of residents and their ratings using the five-and seven-point questionnaires

Questionnaire	5 pt	7 pt
Number of training programs	10	8
Number of residents	119	206
Number of measurement periods that residents participated in^a^	150	241
Median number of ratings per resident per measurement period	7	7
Number of ratings	1264	2115
Male residents (%)	38	41.6
Ratings by year of residency training (%)		
1	12.4	34.1
2	34.9	17.1
3	12.5	4.4
4	22.9	24.3
≥5	17.3	20.1
Number of faculty	175	254
Number of faculty measurement periods^b^	273	354
Median number of ratings per faculty per measurement period	3	5
Number of faculty evaluated by ≥3 residents^c^	205	275
Median number of resident ratings (from faculty rated by ≥3 residents)	4	6

^a, b^A measurement period is a four- to six-week data collection period. Some residents and faculty participated in more than one measurement period from January 2013 to January 2017

^c^Aggregated faculty scores require three or more residents’ ratings to be reliable

Between January 2013 and January 2017, 3379 residents’ ratings evaluated 327 unique teaching faculty; 102 participated using both questionnaires. On average, residents provided 7 ratings (median) per measurement period. Male residents comprised 38 and 41.6% respectively of the five- and seven-point questionnaire sample. Around 50% of the ratings were from residents in their first or second year of training. Ratings on both questionnaires added up to 480 aggregated faculty scores (≥ 3 ratings). The median number of ratings per faculty was 4 and 6 for the five- and seven-point questionnaire, respectively.

### Means, standard deviations and IQRs

Comparisons of means, standard deviations and IQRs are presented in Table 3. Not rescaled seven-point scale ratings showed more variability (expressed in standard deviations and IQRs). IQRs showed that residents used the end-point of the five-point scale more often. In addition, the seven-point scale items showed more spread and room to measure performance change.

**Table 3:** Means, standard deviations and IQRs of residents’ ratings and faculty scores of five-and seven-point questionnaire items

	Residents’ ratings								Aggregated faculty scores							
	5 pt	7 pt		5 pt	7 pt		5 pt	7 pt	5 pt	7 pt		5 pt	7 pt		5 pt	7 pt
Item	M (R)	M	Δ M	SD (R)	SD	Δ SD	IQR	IQR	M (R)	M	Δ M	SD (R)	SD	Δ SD	IQR	IQR
1.	3.98 (5.47)	5.46	.00	.86 (1.29)	1.21	.08^a^	4.00–5.00	5.00–6.00	3.98 (5.47)	5.49	.02	.59 (.88)	.78	.10	3.67–4.33	5.00–6.00
2.	3.95 (5.42)	5.45	.03	.90 (1.35)	1.22	.13	4.00–5.00	5.00–6.00	3.95 (5.43)	4.46	.03	.61 (.92)	.79	.13	3.67–4.33	5.00–6.00
3.	3.95 (5.43)	5.44	.01	.90 (1.35)	1.21	.14	4.00–5.00	5.00–6.00	3.94 (5.41)	5.47	.06	.61 (.92)	.80	.11	3.67–4.33	5.00–6.00
4.	3.74 (5.12)	5.10	.01	.91 (1.37)	1.23	.14^a, b^	3.00–4.00	4.00–6.00	3.71 (5.08)	5.14	.06	.61 (.91)	.83	.08	3.33–4.13	4.67–5.75
5.	4.00 (5.50)	5.56	.07	.90 (1.35)	1.15	.20	4.00–5.00	5.00–6.00	3.98 (5.47)	5.58	.11	.61 (.91)	.79	.12	3.67–4.40	5.17–6.17
6.	4.09 (5.63)	5.78	.15*	.95 (1.43)	1.30	.13	4.00–5.00	5.00–7.00	4.13 (5.69)	5.77	.08	.62 (.93)	.89	.04	3.75–4.57	5.43–6.40
7.	4.29 (5.93)	6.00	.06	.89 (1.33)	1.21	.12^a^	4.00–5.00	6.00–7.00	4.35 (6.02)	6.00	.03	.58 (.86)	.80	.06	4.00–4.70	5.67–6.60
8.	4.34 (6.00)	6.12	.12	.82 (1.22)	1.16	.07^a, b^	4.00–5.00	6.00–7.00	4.38 (6.07)	6.12	.06	.51 (.75)	.76	.00	4.20–4.75	6.00–6.62
9.	3.97 (5.45)	5.40	.05	.84 (1.26)	1.21	.05^a, b^	4.00–4.00	5.00–6.00	3.95 (5.42)	5.45	.03	.54 (.82)	.80	.01	3.67–4.33	5.00–6.00
10.	3.96 (5.44)	5.41	.02	.85 (1.28)	1.22	.06^a^	4.00–5.00	5.00–6.00	3.96 (5.44)	5.46	.02	.56 (.85)	.80	.05	3.67–4.33	5.00–6.00
11.	4.03 (5.55)	5.76	.21*	.79 (1.19)	1.08	.11	4.00–5.00	5.00–6.00	4.06 (5.59)	5.75	.16	.47 (.70)	.65	.05	3.80–4.33	5.50–6.62
12.	4.06 (5.59)	5.84	.25*	.87 (1.30)	1.14	.15	4.00–5.00	5.00–7.00	4.10 (5.66)	5.84	.18	.54 (.81)	.70	.11	3.90–4.40	5.57–6.29
TS.	4.03 (5.54)	5.61	.07	.71 (1.06)	0.99	.07	3.75–4.50	5.17–6.25	4.04 (5.56)	5.63	.06	.49 (.73)	.68	.05	3.85–4.37	5.33–6.10

*p* < .05 after Bonferroni correction

^a^Equal variances could not be assumed

^b^After applying weights to the analysis, equal variances could not be assumed

Mean differences in residents’ ratings were modest for item 6 (*d* = .021, *t*(2456,67) = 2.95, *p* = .039), item 11 (*d* = .026, *t*(3377) = 5.36, *p* < .001), and item 12 (*d* = .033, *t*(3377) = 5.80, *p* < .001), with Bonferroni corrected *p*-values. After applying the calculated weights to the analysis, independent t-tests showed no mean differences for residents’ ratings and aggregated faculty scores. Levene’s test found that equal variances could not be assumed for item 1 (*F* = 15.7*, p* = .009), item 4 (*F* = 24.138*, p* < .001*),* item 7 (*F* = 56.7*, p* < .001), item 8 (*F* = 41.3*, p* < .001), item 9 (*F* = 27.3*, p* < .001), item 10 (*F* = 16.5*, p* = .008), with Bonferroni corrected *p*-values. However, after applying weights, the variances of item 1, 7, and 10 could be assumed equal.

### Frequency distributions and scoring proportions

Table 4 shows the frequencies of the response categories ‘totally disagree’, ‘neutral’ and ‘totally agree’ and the proportions of scores below and above the ‘neutral’ for items of the five- and seven-point questionnaire. Table 4 depicts which frequency distributions were different at *p* < 0.05 for both scales.

**Table 4:** Percentages for similar response categories and percentages below and above the neutral response category for the five- and seven-point questionnaire items

	Totally disagree		Neutral		Totally agree		Below		Above	
Item	5 pt	7 pt	5 pt	7 pt	5 pt	7 pt	5 pt	7 pt	5 pt	7 pt
1.	1.3	.7	15.5	13.4	27.1*	18.5*	6.3	6.2	78.2	80.4
2.	1.9*	.8*	15.3	13.9	26.7*	17.6*	7.4	6.1	77.3	79.9
3.	1.7*	.6*	17.6*	14.0*	28.4*	19.0*	6.8	6.1	75.6*	79.9*
4.	1.5	.9	27.5*	21.8*	20.5*	12.0*	8.5	8.4	64.0*	69.8*
5.	2.0*	.7*	15.5*	10.0*	29.9*	19.0*	6.3	4.9	78.2*	85.2*
6.	2.8*	1.5*	11.6*	5.9*	37.5*	31.8*	7.1	7.1	81.3*	87.0*
7.	2.3	1.3	8.0*	4.5*	48.5*	39.7*	4.7	5.2	87.3*	90.3*
8.	1.3	1.0	6.6*	3.8*	49.3	45.5	3.9	4.3	89.5	91.8
9.	1.3	.7	15.1	12.6	24.9*	16.9*	6.0	7.4	78.9	80.0
10	1.4	.9	17.0*	13.0*	25.6*	17.0*	5.7	7.0	77.3	80.0
11.	1.3*	.5*	13.6*	7.2*	26.3	24.2	4.1	3.8	82.3*	88.9*
12.	1.6	.7	12.7*	7.1*	31.8	29.9	5.8	4.5	81.5*	88.3*

* Percentages of both scales differ with *p* < 0.05 (Bonferroni correction included)

Chi-square tests showed that except for item 12, frequencies of the ‘totally agree’ category differed for all items of both questionnaires. Also, except for item 1, 2, and 9, frequencies of the ‘neutral’ category differed for all items of both questionnaires. Differences in frequencies of the category ‘totally disagree’ of item 2, 3, 5, 6, 11 were small or uncertain for both questionnaires.

Concerning the proportion of ratings below the ‘neutral’ category, no substantial differences were found. However, for item 3, 4, 5, 6, 7, 11, and 12, proportions above the ‘neutral’ category were different. Residents used the response categories above the ‘neutral’ category on the seven-point scale more often. Frequencies of all response categories of the seven-point scale were lower compared to the five-point scale.

## Discussion

### Main findings

This study compared the variability of residents’ ratings of faculty’s teaching performance measured by validated five- and seven-point response scales. Residents used the additional response categories of the seven-point scale, especially to differentiate between positive performances. Seven-point scale ratings were more evenly distributed and had a smaller ceiling effect, also when aggregated to faculty scores. After rescaling, means and standard deviations of ratings on both scales showed no substantial differences.

### Explanation of main findings

In line with our expectations [14, 16], residents used the additional response categories of the seven-point scale. Also, as expected [12, 13], we found no substantial differences in means and standard deviations of aggregated faculty scores. This indicates that, while both scales provide (almost) similar teaching performance outcomes, the seven-point scale offers more room for residents to differentiate between supervisors’ performance. Still, without adjusting for non-randomised samples of residents’ ratings, means of three items were statistically different, although effect sizes were small.

Concerning frequency distributions, all categories of the seven-point scale were used less frequently than those of the five-point scale. Differences in frequencies were most substantial for the categories ‘totally agree’ and ‘neutral’. For some items, the category ‘totally disagree’ was rated less frequently on the seven-point scale than on the five-point scale.

Hassel et al. [17] identified a higher proportion of students as ‘above expectations’ and a smaller proportion as ‘of potential concern’ using longer response scales. In this study, we also found a higher proportion of scores above the ‘neutral’ category, but proportions below the ‘neutral’ category did not differ substantially. Hence, on the seven-point scale, residents used the options ‘disagree’, ‘somewhat disagree’ ‘somewhat agree’ and ‘agree’ to differentiate between performances.

Residents and faculty members might value the additional response categories provided by the seven-point scale. When residents feel like the response scale reflects their perceptions more accurately, they might perceive the questionnaire as more valid and user-friendly [16, 21]. Also, residents may value the opportunity to offer more nuanced responses in follow-up discussions about teaching performance feedback. Residents and faculty members generally discuss feedback measured by the SETQ system in facilitated meetings [36]. In such meetings, more response categories provide more specific directions for future improvement, especially when discussing item-level ratings. The need for specific performance information was previously found to be the main reason for faculty members to discuss the feedback generated by the SETQ system [23].

Furthermore, residents rated the response category ‘totally agree’ less frequently on the seven-point scale as compared to the five-point scale, which implies a smaller ceiling effect, contributing to the questionnaires’ ability to measure faculty’s teaching performance improvement. Faculty members might value the improved ability to measure change, as they often have much work experience and room for performance improvement is relatively limited [14]. Based on indicators of variance, we expect the seven-point scale to be more sensitive to performance change [14, 16, 37].

Logically, residents and faculty members might have different evaluation preferences. Some residents may find a five-point scale easier to use than a seven-point scale, and faculty members could appraise the resulting feedback as more straightforward. However, if they prefer more response categories for nuanced evaluation, or more specific feedback respectively, using a seven-point scale seems beneficial.

Critically evaluating and choosing response scales may improve the practice of faculty’s teaching performance evaluation. However, the translation from feedback to actual improvement depends on many aspects, for example, whether faculty members discuss the provided feedback. For effective use of feedback during these discussions, positive attitudes towards receiving feedback are crucial [36, 38, 39]. Therefore, we agree with others that enhancing teaching performance requires an integral approach, including measures with good psychometric qualities and a culture in which it is safe to discuss feedback openly and constructively [20, 38].

### Limitations and strengths of this study

This study contributed to the limited knowledge of how the number of response categories affects residents’ ratings of faculty’s teaching performance [9, 17, 32]. Our findings should be considered in light of some limitations and strengths of this study.

First, residents were not randomly assigned to the five- and seven-point questionnaires. Descriptive statistics showed some differences in the characteristics of respondents and their ratings. We cannot rule out that sample differences influenced our results. However, a strength of this study was the relatively large number of teaching performance ratings from Dutch anaesthesiology training programs. Besides that, we compared (rescaled) means and standard deviations of ratings measured by both scales with a weighting score to control for non-randomly assigned samples. Applying a weighting score led to small differences in our results. These differences invigorated our more general finding that the means and standard deviations of ratings from both scales did not differ substantially.

Second, the five- and seven-point questionnaires were not entirely similar. For example, the total number of items and their sequential order differed somewhat. Although we compared identical items, we cannot rule out the possibility of small variations in questionnaire design affecting the observed responses [40, 41]. Still, in developing both questionnaires, we tried to comply with the rule of general questions preceding specific questions [41], minimalizing the effect of the number and sequence of items on the results in this study.

With these limitations in mind, our findings can contribute to the knowledge base on choice and implications of response scales for research and practice in teaching performance evaluation and improvement.

### Implications for research and practice

More research is needed on how response scales affect residents’ ratings of faculty’s teaching performance and performance improvement. First, future research should aim at replicating our results in controlled settings using a wider variety of scale formats. Longitudinal research designs could validate inferences about response scales’ abilities to measure residents’ perceptions and faculty’s performance change. Additionally, longitudinal studies with the same cohorts of residents and faculty can separate signal from noise and assess whether seven-point scales reflect residents’ perceptions more adequately than five-point scales. Furthermore, such designs can determine response scales’ sensitivity to measure performance change over time. Second, to optimise faculty’s teaching performance evaluation, it would be worthwhile to investigate the effect of the number of response categories along with other scale properties on teaching performance ratings, such as labelling of response categories [42, 43] or changing the ratio between positive or negative response categories [44].

In addition to such response scale adjustments, researchers should evaluate the experiences and preferences of respondents [15]. Research on faculty’s experiences with feedback based on different response scales could further clarify implications for teaching performance improvement.

In terms of practice, residents and faculty members should critically evaluate whether response scales fit their preferences and goals. If their preference is to add nuance and specificity to teaching performance feedback, seven-point scales would be preferable to five-point scales.

## Conclusions

This study showed that five- and seven-point response scales yield similar outcomes when evaluating faculty’s teaching performance. However, residents used the additional response categories of the seven-point scale to differentiate faculty members’ performance. Also, the seven-point scale data were more optimally distributed. Switching to the seven-point response scale could be of benefit to the resulting feedback, the user-friendliness of the evaluation tool, and the ability to measure performance change.

## Supplementary information

**Additional file 1.**

## Data Availability

Data from this study are not publicly available. Inquiries about potential research collaboration can be directed to the corresponding author.

## Abbreviations

SETQ: System for Evaluation of Teaching Qualities
IQR: Interquartile Range
TS: Total performance score
