# Supplementary material for: Variability of residents’ ratings of faculty’s teaching performance measured by five- and seven-point response scales
Source: BMC Med Educ. 2020 Sep 22;20:325. doi: 10.1186/s12909-020-02244-9 (PMC7510269; doi:10.1186/s12909-020-02244-9)
Supplement: Supplementary file 1 — Additional file 1. [file 12909_2020_2244_MOESM1_ESM.docx]

**Appendix**

| During my anesthesiology residency training,  this attending/ supervisor generally …. | | 1 = totally disagree  2 = disagree  3 = neutral  4 = agree  5 = totally agree | Not applicable/  I can not judge’ |
| --- | --- | --- | --- |
| Learning Climate | | | |
|  | Encourages residents to participate actively in discussions | **1 🞎 2 🞎 3 🞎 4 🞎 5 🞎** | **🞎** |
|  | Stimulates residents to bring up problems | **1 🞎 2 🞎 3 🞎 4 🞎 5 🞎** | **🞎** |
|  | Motivates residents to study further | **1 🞎 2 🞎 3 🞎 4 🞎 5 🞎** | **🞎** |
|  | Stimulates residents to keep up with the literature | **1 🞎 2 🞎 3 🞎 4 🞎 5 🞎** | **🞎** |
|  | Prepares well for teaching presentations and meetings | **1 🞎 2 🞎 3 🞎 4 🞎 5 🞎** | **🞎** |
|  | Teaches residents the full spectrum of postoperative care | **1 🞎 2 🞎 3 🞎 4 🞎 5 🞎** | **🞎** |
| Professional attitude towards residents | | | |
|  | Listens attentively to residents | **1 🞎 2 🞎 3 🞎 4 🞎 5 🞎** | **🞎** |
|  | Is respectful towards residents | **1 🞎 2 🞎 3 🞎 4 🞎 5 🞎** | **🞎** |
|  | Is easily approachable during on-calls | **1 🞎 2 🞎 3 🞎 4 🞎 5 🞎** | **🞎** |
|  | Easily approachable for discussions during pain clinic | **1 🞎 2 🞎 3 🞎 4 🞎 5 🞎** | **🞎** |
| Communication of goals | | | |
|  | States learning goals clearly | **1 🞎 2 🞎 3 🞎 4 🞎 5 🞎** | **🞎** |
|  | States relevant goals | **1 🞎 2 🞎 3 🞎 4 🞎 5 🞎** | **🞎** |
|  | Prioritizes learning goals | **1 🞎 2 🞎 3 🞎 4 🞎 5 🞎** | **🞎** |
|  | Repeats stated learning goals periodically | **1 🞎 2 🞎 3 🞎 4 🞎 5 🞎** | **🞎** |
| Evaluation of residents | |  |  |
|  | Evaluates residents’ specialty knowledge regularly | **1 🞎 2 🞎 3 🞎 4 🞎 5 🞎** | **🞎** |
|  | Evaluates residents’ analytical abilities regularly | **1 🞎 2 🞎 3 🞎 4 🞎 5 🞎** | **🞎** |
|  | Evaluates residents’ application of knowledge to specific patients regularly | **1 🞎 2 🞎 3 🞎 4 🞎 5 🞎** | **🞎** |
|  | Evaluates residents’ medical skills regularly | **1 🞎 2 🞎 3 🞎 4 🞎 5 🞎** | **🞎** |
| Feedback to residents | |  |  |
|  | Regularly gives positive feedback to residents | **1 🞎 2 🞎 3 🞎 4 🞎 5 🞎** | **🞎** |
|  | Gives corrective feedback to residents | **1 🞎 2 🞎 3 🞎 4 🞎 5 🞎** | **🞎** |
|  | Explains why residents are incorrect | **1 🞎 2 🞎 3 🞎 4 🞎 5 🞎** | **🞎** |
|  | Offers suggestions for improvement | **1 🞎 2 🞎 3 🞎 4 🞎 5 🞎** | **🞎** |
|  | Overall, I rate this attending’s/supervisor’s overall teaching performance as | **1 🞎 2 🞎 3 🞎 4 🞎 5 🞎** |  |

| During my anesthesiology residency training,  this attending/ supervisor generally …. | | Please rate this attending/supervisor on a 7-point scale where:   1 = totally disagree  2 = disagree  3 = somewhat disagree  4 = neutral  5 = somewhat agree  6 = agree  7 = totally agree | | Not applicable/  I can not judge’ |
| --- | --- | --- | --- | --- |
| Learning Climate | | | | |
|  | Encourages residents to participate actively in discussions | **1 🞎 2 🞎 3 🞎4 🞎5 🞎 6🞎 7🞎** | |  |
|  | Stimulates residents to bring up problems | **1 🞎 2 🞎 3 🞎4 🞎5 🞎 6🞎 7🞎** | |  |
|  | Motivates residents to study further | **1 🞎 2 🞎 3 🞎4 🞎5 🞎 6🞎 7🞎** | |  |
|  | Stimulates residents to keep up with the literature | **1 🞎 2 🞎 3 🞎4 🞎5 🞎 6🞎 7🞎** | |  |
|  | Prepares well for teaching presentations and meetings | **1 🞎 2 🞎 3 🞎4 🞎5 🞎 6🞎 7🞎** | | **🞎** |
|  | Teaches residents the full spectrum of perioperative care |  | |  |
| Professional Attitude Towards Residents | | | | |
|  | Listens attentively to residents | **1 🞎 2 🞎 3 🞎4 🞎5 🞎 6🞎 7🞎** | |  |
|  | Is respectful towards residents | **1 🞎 2 🞎 3 🞎4 🞎5 🞎 6🞎 7🞎** | |  |
|  | Is easily approachable during on-calls | **1 🞎 2 🞎 3 🞎4 🞎5 🞎 6🞎 7🞎** | | **🞎** |
|  | Is easily approachable for discussion during routine daytime work | **1 🞎 2 🞎 3 🞎4 🞎5 🞎 6🞎 7🞎** | |  |
| Learner centeredness | | | | |
|  | Clarifies learning goals for the learning session | **1 🞎 2 🞎 3 🞎4 🞎5 🞎 6🞎 7🞎** | |  |
|  | Matches residents’ and supervisor’s learning expectations | **1 🞎 2 🞎 3 🞎4 🞎5 🞎 6🞎 7🞎** | |  |
|  | Provides residents with responsibility based on their abilities | **1 🞎 2 🞎 3 🞎4 🞎5 🞎 6🞎 7🞎** | |  |
|  | Teaches residents how to deal with competing personal/professional demands | **1 🞎 2 🞎 3 🞎4 🞎5 🞎 6🞎 7🞎** | |  |
| Evaluation | |  | |  |
|  | Evaluates residents’ specialty knowledge regularly | **1 🞎 2 🞎 3 🞎4 🞎5 🞎 6🞎 7🞎** | |  |
|  | Evaluates residents’ analytical abilities regularly | **1 🞎 2 🞎 3 🞎4 🞎5 🞎 6🞎 7🞎** | |  |
|  | Evaluates residents’ application of knowledge in daily practice regularly | **1 🞎 2 🞎 3 🞎4 🞎5 🞎 6🞎 7🞎** | |  |
|  | Evaluates residents’ procedural skills regularly | **1 🞎 2 🞎 3 🞎4 🞎5 🞎 6🞎 7🞎** | |  |
| Feedback to Residents | |  | |  |
|  | Gives positive feedback to residents | **1 🞎 2 🞎 3 🞎4 🞎5 🞎 6🞎 7🞎** | |  |
|  | Gives corrective feedback to residents | **1 🞎 2 🞎 3 🞎4 🞎5 🞎 6🞎 7🞎** | |  |
|  | Explains why residents are (in)correct | **1 🞎 2 🞎 3 🞎4 🞎5 🞎 6🞎 7🞎** | |  |
|  | Offers suggestions for improvement | **1 🞎 2 🞎 3 🞎4 🞎5 🞎 6🞎 7🞎** | |  |
|  | Overall, I rate this attending’s/supervisor’s overall teaching performance as | | **1 🞎 2 🞎 3 🞎4 🞎5 🞎 6🞎 7🞎 8🞎 9🞎 10🞎** | |

* Commercial use is not allowed. Please contact the corresponding author for academic use of

the questionnaires.
